# A bisazobenzene crosslinker that isomerizes with visible light

**DOI:** 10.3762/bjoc.8.246

**Published:** 2012-12-14

**Authors:** Subhas Samanta, Harris I Qureshi, G Andrew Woolley

**Affiliations:** 1 Department of Chemistry, University of Toronto, 80 St. George St., Toronto, ON, M5S 3H6, Canada; telephone: (416) 978-0675, fax: (416) 978-8775

**Keywords:** azobenzene, molecular switches, switch, photo-control, visible

## Abstract

**Background:** Large conformational and functional changes of azobenzene-modified biomolecules require longer azobenzene derivatives that undergo large end-to-end distance changes upon photoisomerization. In addition, isomerization that occurs with visible rather than UV irradiation is preferred for biological applications.

**Results:** We report the synthesis and characterization of a new crosslinker in which a central piperazine unit links two azobenzene chromophores. Molecular modeling indicates that this crosslinker can undergo a large change in end-to-end distance upon *trans*,*trans* to *cis*,*cis* isomerization. Photochemical characterization indicates that it does isomerize with visible light (violet to blue wavelengths). However, the thermal relaxation rate of this crosslinker is rather high (τ_½_ ~ 1 s in aqueous buffer at neutral pH) so that it is difficult to produce large fractions of the *cis*,*cis*-species without very bright light sources.

**Conclusion:** While cis-lifetimes may be longer when the crosslinker is attached to a biomolecule, it appears the *para*-piperazine unit may be best suited for applications where rapid thermal relaxation is required.

## Introduction

**Scheme 1:**
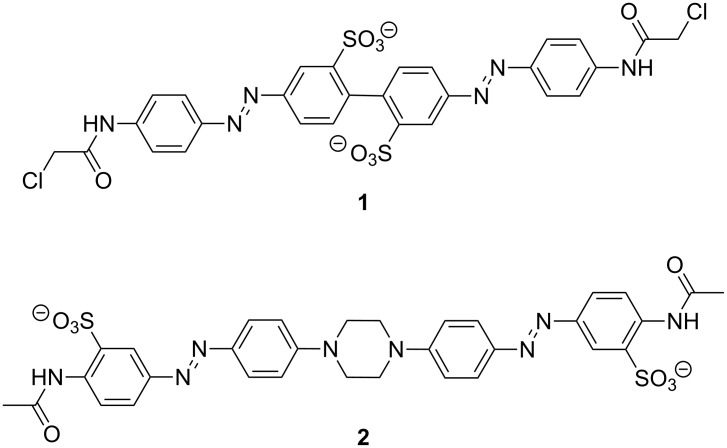
Bisazobenzene derivatives.

Azobenzene derivatives have been used for reversible manipulation of biological targets, including peptide and protein structure and function [1–12], enzyme activities [13–17], oligonucleotide functions [18–20], and ion-channel activities [21–23]. A quantitative analysis of the effects of crosslinkers on protein folding led to the conclusion that photocontrol of folding is best achieved by using rigid crosslinkers that undergo a large change in end-to-end distance upon photoisomerization [6]. Previously, we reported the design and synthesis of the rigid bisazobenzene crosslinker BPDBS, (4,4'-bis(4-(2-chloroacetamido)phenyl)diazenylbiphenyl-2,2'-disulfonate) (**1**, Scheme 1) [24]. This photoswitch can produce a minimum ~5 Å and a maximum ~23 Å end-to-end distance change upon *trans*–*cis* isomerization. It has a high absorption coefficient (45–60,000 M^−1^cm^−1^) and can produce up to ~80% *trans*–*cis* isomerization under favorable conditions. The suitability of this photoswitch for biological systems would be enhanced if the wavelengths required for photoisomerization were longer, so that UV light was not required. UV light is highly scattered by biological samples and can be a toxic stimulus [25–26]. In an attempt to red-shift the switching wavelength while maintaining a large end-to-end distance change upon photoisomerization, we designed compound **2** (Scheme 1). The central piperazine unit in this structure was expected to disconnect the π-delocalization between the two azo chromophores completely, while providing N-centered lone pairs to extend the π-system of each independent azo unit. In this manner, independent photoswitching behavior of the two units at wavelengths shifted towards the visible range was expected to occur.

## Results and Discussion

**Scheme 2:**
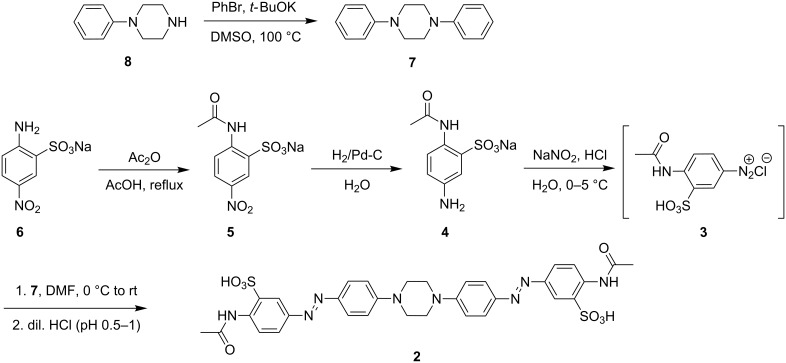
Synthetic route for the preparation of bisazobenzene derivative **2**.

The *p*-diacetamido piperazine-linked bisazobenzene derivative **2** was prepared by a double diazo-coupling reaction between the donor 1,4-diphenylpiperazine (**7**) and the acceptor 4-acetamido-3-sulfobenzenediazonium chloride (**3**), which was synthesized in situ by diazotization of sodium 2-acetamido-5-aminobenzenesulfonate (**4**) using sodium nitrite with HCl (Scheme 2). Compound **4** was prepared by hydrogenation with H_2_/Pd-C from sodium 2-acetamido-5-nitrobenzenesulfonate (**5**), which was obtained from commercially available sodium 2-amino-5-nitrobenzenesulfonate (**6**) by acetylation using acetic anhydride. 1,4-Diphenylpiperazine (**7**) was synthesized by a cross-coupling reaction of bromobenzene with 1-phenylpiperazine in the presence of *t*-BuOK.

**Figure 1:**
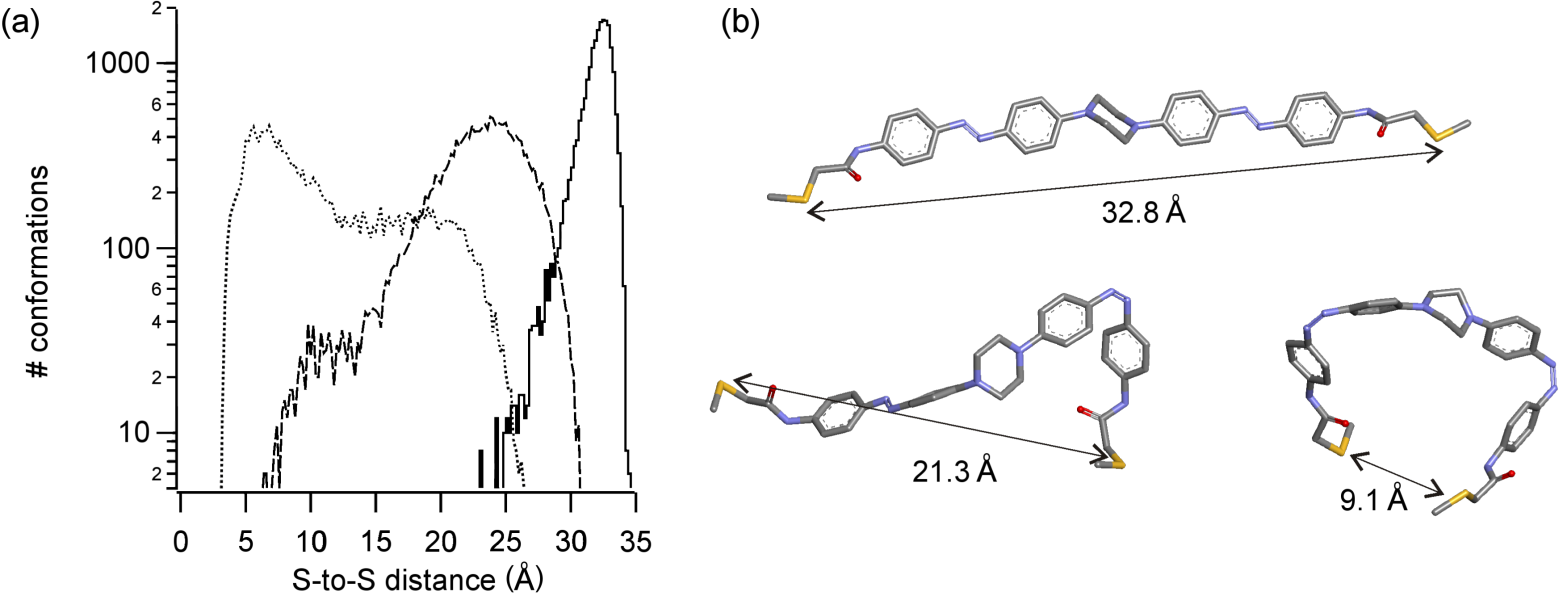
(a) Histograms showing the relative populations of conformations having the sulfur-to-sulfur distances indicated. (b) Examples of *trans*,*trans*, *trans*,*cis* and *cis*,*cis* conformations with the S–S distances indicated.

As with previous crosslinkers, attachment to a target peptide or protein would involve replacement of the two terminal acetamido units with chloro- or iodoacetamide units to enable reaction with Cys residues [24]. The end-to-end distance changes expected upon photoisomerization of the resulting crosslink were estimated by molecular dynamics methods. Figure 1 shows models of compound **2** in *trans*,*trans*, *trans*,*cis*, and *cis*,*cis*-isomeric states. These simulations indicate that the *trans*,*trans* and *cis*,*cis*-states give well-separated end-to-end distance changes. The *trans*,*trans*-isomer has a preferred end-to-end distance of 32.8 Å but some flexibility in the piperazine linkage permits distances between 25 and 35 Å. The *cis*,*cis*-isomer permits distances between ~4 and 25 Å. The *trans*,*cis*-isomers produce an intermediate range of distances between 7 and 30 Å, which overlaps with both *trans*,*trans* and *cis*,*cis*-isomers. This rather large and overlapping range of distances for the *trans*,*cis*-species is problematic from the point of view of photocontrol since a large light-driven change in the end-to-end distribution will require very efficient photoisomerization to the *cis*,*cis*-species. We were, therefore, interested in the photochemical behavior of **2**.

**Figure 2:**
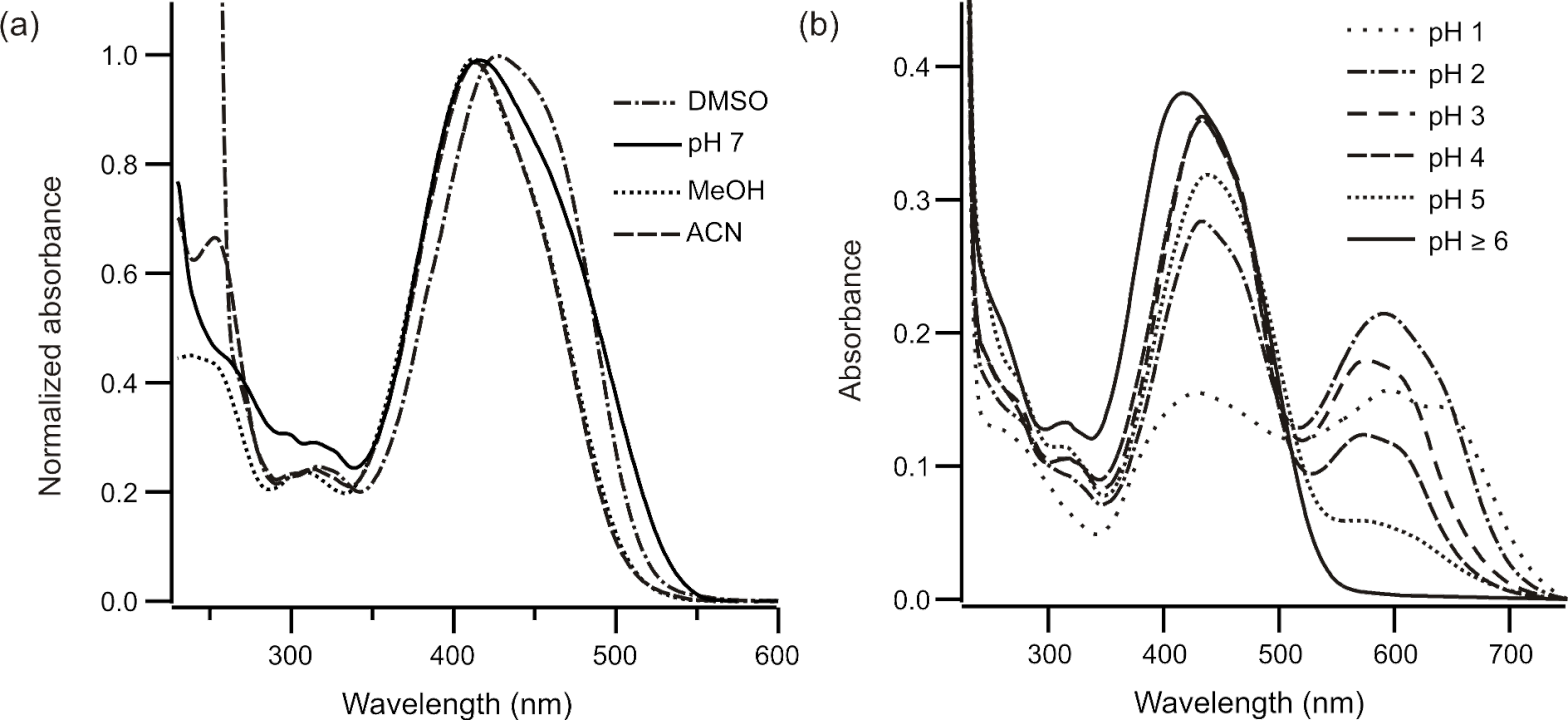
(a) Normalized UV–vis absorption spectra of **2** in DMSO, acetonitrile, MeOH and sodium phosphate buffer (pH 7.0). (b) UV–vis absorption spectra of **2** (~10 μM) in aqueous buffer at different pHs (indicated).

Figure 2 shows UV–vis absorbance spectra of **2** in the dark-adapted *trans,trans*-form in various solvents. As expected, the main absorbance band (π–π* transition) is red-shifted compared to BPDBS with a maximum near 420 nm. The molar extinction coefficient for **2** was determined by ^1^H NMR coupled with UV–vis absorption spectra, to be 66,400 in DMSO at 428 nm, such that on a molar basis, the photoswitch is intensely colored.

The spectra in aqueous solutions are pH dependent below pH 6.0 (Figure 2b). A band corresponding to the azonium species [27] near 600 nm is evident at pH 5 and this does not appear to change upon irradiation. At lower pH values the spectrum undergoes further changes with enhanced absorbance from 530–700 nm consistent with the formation of a doubly protonated bisazonium species. This species is expected to be in equilibrium with the corresponding bisammonium species [27].

**Figure 3:**
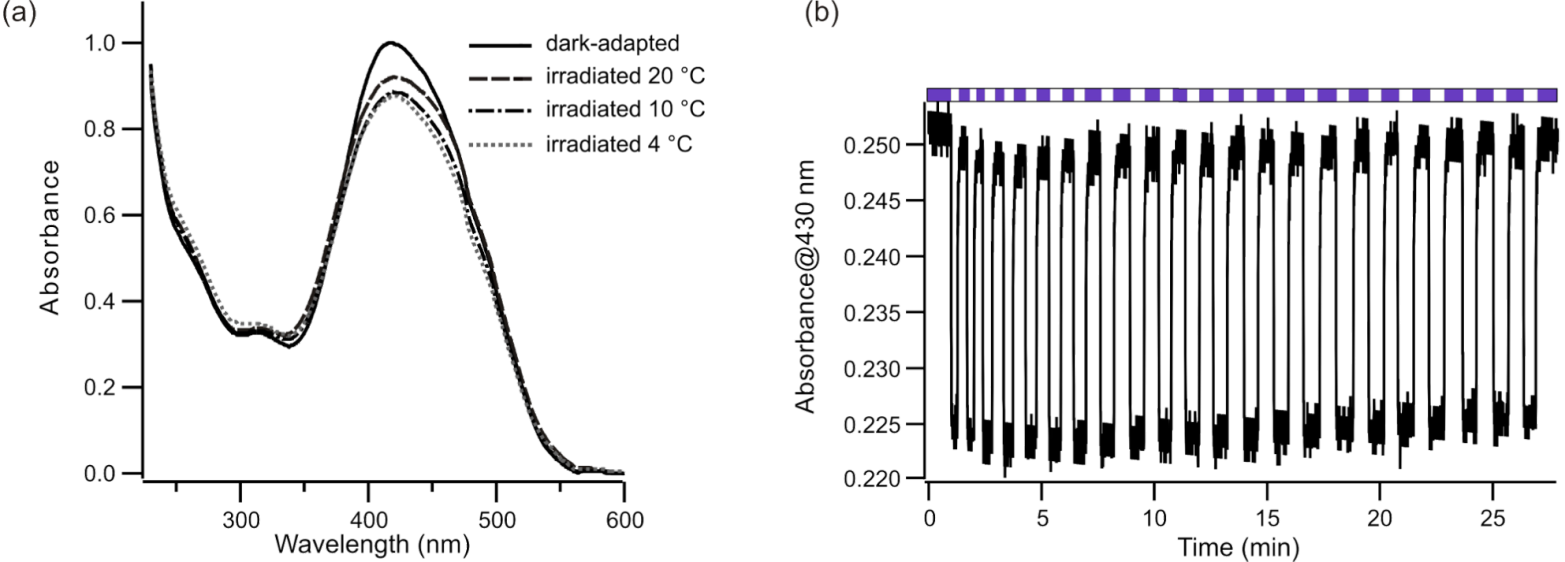
(a) Photoswitching of **2** with violet light (407–410 nm) at different temperatures (4, 10 and 20 °C) in 25 mM sodium phosphate buffer at pH 8. (b) Multiple rounds of photoswitching of **2** with alternating violet light (violet bars) and dark adaption (white bars) in 25 mM phosphate buffer, pH 8, at 10 °C.

Irradiation of **2** in DMSO and in acetonitrile caused only small changes in the spectra indicating rapid thermal reversal. However, switching in aqueous solutions was measurable (Figure 3). The half-life of the irradiated isomers of **2** in phosphate buffer at 4 °C was measured to be 1.5 ± 0.3 s.

Numerous cycles of photoisomerization could be carried out without noticeable photobleaching (Figure 3b). The relatively rapid thermal relaxation, however, means that the overall *trans* versus *cis*-isomer ratio is small, and the fraction of the *cis*,*cis*-species is low. While *cis* lifetimes may be longer when the crosslinker is attached to a biomolecule, it is likely that this crosslink will predominantly cycle between the *trans*,*trans* and *trans*,*cis*-species under low irradiation intensities. These species have overlapping end-to-end distances and would not be expected to drive significant conformational change in a single attached biomolecule [6]. However, high (e.g., pulsed) light intensities may be expected to populate the *cis*,*cis*-state and may permit pulsed-light-driven conformational changes. It is possible that further substitution, particularly in positions *ortho* to the azo units may slow down the thermal relaxation rates and make production of a large fraction of the *cis*,*cis*-species possible [28]. Alternatively, azobenzene-based switches with rapid thermal relaxation times are preferred in materials-based applications where they can lead to light-driven changes in polymer properties. Bisazobenzene-based compounds analogous to **2** may be useful in this context [29–33].

## Experimental

**General aspects:** All commercial materials (solvents, reagents and substrates) were used as received. NMR spectra were recorded either on Varian Vnmr-S 400 MHz, Varian Mercury 400 MHz or Agilent DD2 500 MHz spectrometers. Silica gel of particle size 40–63 μm from Silicycle Chemical Division was used for column chromatography.

**Synthesis of 1,4-diphenylpiperazine (7)** [34]: A mixture of 1-phenylpiperazine (**8**, 2.0 g, 12.3 mmol), bromobenzene (1.9 g, 12.1 mmol) and *t*-BuOK (2.7 g, 24.11 mmol) in anhydrous DMSO (25 mL) was heated at 100 °C for 2 h. The reaction was quenched with water and extracted with dichloromethane. The combined organic layer was dried over anhydrous sodium sulfate and concentrated. The crude oil was subjected to silica-gel column chromatography to afford 1,4-diphenylpiperazine (**7**, 1.2 g, yield 41%) as a colorless solid. ^1^H NMR (400 MHz, CDCl_3_) δ 3.34 (s, 8H), 6.89 (t, *J* = 7.6 Hz, 2H), 6.99 (d, *J* = 8.0 Hz, 4H), 7.29 (t, *J* = 7.6 Hz, 4H) ppm.

**Synthesis of sodium 2-acetamido-5-nitrobenzenesulfonate (5):** To sodium 2-amino-5-nitrobenzenesulfonate (**6**, 4.0 g, 16.6 mmol) in acetic acid (40 mL) was added acetic anhydride (2.6 g, 24.8 mmol) and the solution was heated under reflux for 4 h. After cooling to rt, the precipitate was filtered, washed with hot acetic acid and then ether, and dried under high vacuum. The crude product was resuspended in hot ethanol, filtered and dried to obtain sodium 2-acetamido-5-nitrobenzenesulfonate (**5**, 4.3 g, yield 90%) as a colorless solid. ^1^H NMR (400 MHz, DMSO-*d*_6_) δ 2.13 (s, 3H), 8.21 (dd, *J*_1_ = 9.2 Hz, *J*_2_ = 2.8 Hz, 1H), 8.43 (d, *J* = 2.8 Hz, 1H), 8.55 (d, *J* = 9.2 Hz, 1H), 10.66 (br s, NH) ppm; ^13^C NMR (100 MHz, DMSO-*d*_6_) δ 25.7, 120.2, 123.1, 126.2, 135.9, 141.6, 141.8, 169.3 ppm; HRMS–ESI (*m*/*z*): [M − H]^−^ calcd for C_8_H_7_N_2_O_6_S, 259.2174; found, 259.2172.

**Synthesis of sodium 2-acetamido-5-aminobenzenesulfonate (4)** [35]: To a solution of sodium 2-acetamido-5-nitrobenzenesulfonate (**5**, 4.2 g, 14.9 mmol) in water (100 mL) contained in a double-necked round-bottom flask was added 10% Pd/C (0.3 g) under nitrogen gas atmosphere. A filled hydrogen balloon was then connected to one neck and the reaction vessel was thoroughly degassed and purged with hydrogen gas. The reaction mixture was vigorously stirred under hydrogen-balloon pressure at rt for 14 h. The reaction mixture was filtered through a celite pad, and the water was removed under vacuum. The crude product was resuspended in hot ethanol, filtered and dried to obtain sodium 2-acetamido-5-aminobenzenesulfonate (**4**, 3.6 g, yield 97%) as a colorless solid. ^1^H NMR (400 MHz, DMSO-*d*_6_) δ 1.96 (s, 3H), 4.91 (br, NH_2_), 6.46 (dd, *J*_1_ = 8.4 Hz, *J*_2_ = 2.8 Hz, 1H), 6.95 (d, *J* = 2.8 Hz, 1H), 7.88 (d, *J* = 8.4 Hz, 1H), 9.98 (br, NH) ppm.

**Synthesis of *p*-diacetamido piperazine-linked bisazobenzene, 5,5'-((piperazine-1,4-diylbis(4,1-phenylene))bis(diazene-2,1-diyl))bis(2-acetamidobenzenesulfonic acid) (2):** An ice-cold mixture of sodium 2-acetamido-5-aminobenzenesulfonate (**4**, 1.6 g, 6.3 mmol) and sodium nitrite (0.47 g, 6.8 mmol) in water (30 mL) was added dropwise to a mixture of concentrated hydrochloric acid (2.8 mL) and ice (30.0 g). The resulting mixture was stirred at 0–5 °C for 1 h. To this diazonium solution was added dropwise an ice-cold solution of 1,4-diphenylpiperazine (**7**, 0.5 g, 2.1 mmol) in DMF (10 mL). After stirring for 2 h at 0–5 °C, it was stirred at room temperature for 24 h. The reaction was made acidic (pH 1 to 0.5), filtered and the residue was washed with cold water. The crude product was subjected to a short silica gel column chromatography to isolate *p*-diacetamido piperazine-linked bisazobenzene **2** (108.0 mg, yield 7%), which was further HPLC purified on a semipreparative SB-C8 column with a linear solvent gradient of 10–75% acetonitrile/H_2_O (containing 0.1% trifluoroacetic acid) over a course of 25 min; the product was eluted at 54.5% acetonitrile. ^1^H NMR (400 MHz, DMSO-*d*_6_) δ 2.08 (s, 6H), 3.54 (s, 8H), 7.11 (d, *J* = 9.2 Hz, 4H), 7.77–7.81 (m, 6H), 8.09 (d, *J* = 2.4 Hz, 2H), 8.45 (d, *J* = 8.8 Hz, 2H), 10.58 (br, NH) ppm; ^13^C NMR (125 MHz, DMSO-*d*_6_) δ 25.5, 47.0, 114.6, 119.5, 120.1, 124.8, 125.6, 136.2, 137.1, 144.6, 147.1, 152.9, 168.3 ppm; HRMS–ESI (*m*/*z*): [M − H]^−^ calcd for C_32_H_31_N_8_O_8_S_2_, 719.1711; found, 719.1711.

**UV–vis spectra and photoisomerization**: UV–vis absorption spectra were obtained using either a Perkin-Elmer Lambda 25 spectrophotometer or using a diode array UV–vis spectrophotometer (Ocean Optics Inc., USB4000) coupled to a temperature-controlled cuvette holder (Quantum Northwest, Inc. Spokane, WA, USA). The latter arrangement was used to measure thermal relaxation rates, and steady-state spectra under UV–vis illumination. For steady-state spectra, typically, a 5–10 µM solution of **2** in 25 mM sodium phosphate buffer (pH 8.0), contained in a 1 cm path length quartz cuvette in the temperature-controlled cuvette holder, was irradiated (at 90^o^ to the measuring beam) with LEDs emitting at 407–410 nm (~460 mW, LEDengin LZ1-10UA05) at 4, 10 and 20 °C for 2 min until no further decrease in absorbance was observed, and under this steady-state-irradiation condition the spectra were recorded.

Rates of thermal reversion of the irradiated samples were measured at 4 °C by monitoring absorbance at 425 nm with time. All curves were fitted to mono-exponential decay kinetics. The light used for the absorbance measurement was of sufficiently low intensity to cause negligible isomerization.

To determine the molar extinction coefficient ^1^H NMR spectra of a solution of **2** in DMSO-*d*_6_ containing a known concentration of 1,2-dichloroethane (DCE) as an internal reference was recorded. The NMR solution was later diluted in DMSO and the UV–vis spectrum was recorded to calculate the molar extinction coefficient.

**Molecular modeling**: Models of *trans*,*trans*, *trans*,*cis* and *cis*,*cis*-crosslinkers were built by using HyperChem (v.8, Hypercube Inc.) with the linker terminated with methyl groups representing the β-carbon of Cys in the cross-linked peptide, and minimized by using the Amber99 force field. Restraints were added to the azo-bond for the *trans*,*cis* and *cis*,*cis*-isomers (force constant 16). Molecular dynamics runs were performed in vacuo, essentially as described previously [36], with a distance-dependent dielectric constant, and 1–4 scale factors of 0.833 for electrostatic and 0.5 for van der Waals interactions, a step size of 1 fs and 300 K as the simulation temperature. Trajectories were analyzed to verify that numerous torsion-angle changes occurred for all single bonds during the course of the simulation to ensure that conformational space was adequately sampled. Histograms of all points were then produced for the S–S distance during the full set of simulations for each isomer.

## Supporting Information

File 1 NMR spectra for compounds **2**, **4**, **5** and **7**.
