# Supplementary material for: A bisazobenzene crosslinker that isomerizes with visible light
Source: Beilstein J Org Chem. 2012 Dec 14;8:2184–90. doi: 10.3762/bjoc.8.246 (PMC3554325; doi:10.3762/bjoc.8.246)
Supplement: File 1 — NMR spectra for compounds 2, 4, 5 and 7. [file Beilstein_J_Org_Chem-08-2184-s001.pdf]

Supporting Information

for

A bisazobenzene crosslinker that isomerizes with visible light

Subhas Samanta, Harris I. Qureshi and G. Andrew Woolley*

Address: Department of Chemistry, University of Toronto, 80 St. George St., Toronto, ON, M5S 3H6, Canada; telephone: (416) 978-0675, fax: (416) 978-8775

Email: awoolley@chem.utoronto.ca

*Corresponding author

NMR spectra for compounds 2, 4, 5 and 7

^{1}H NMR spectrum of 1,4-diphenylpiperazine (**7**) S2

^{1}H and ^{13}C NMR spectra of sodium 2-acetamido-5-nitrobenzenesulfonate (**5**) S3

^{1}H NMR spectrum of sodium 2-acetamido-5-aminobenzenesulfonate (**4**) S4

^{1}H and ^{13}C NMR spectra of 5,5'-(piperazine-1,4-diylbis(4,1-phenylene))bis(diazene-2,1-diyl)bis(2-acetamidobenzenesulfonic acid) (**2**) S5

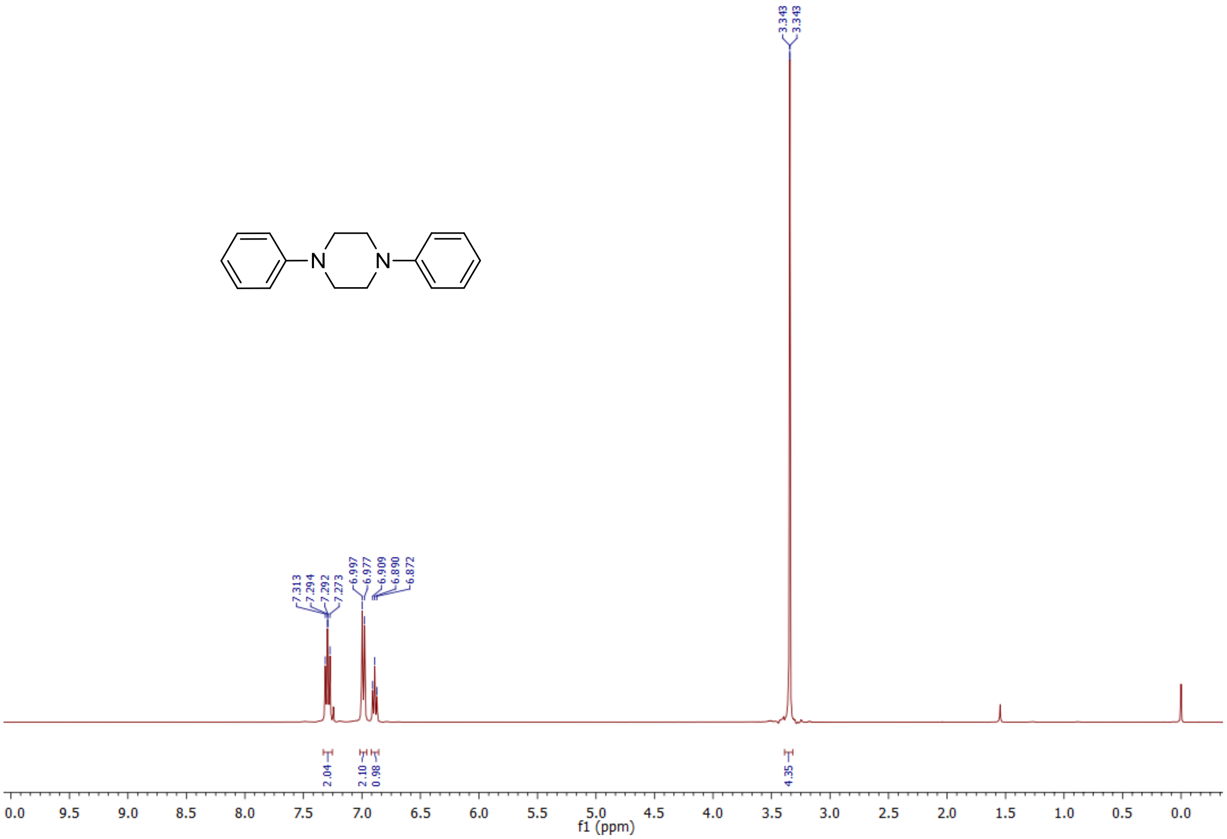

Figure S1: ^{1}H NMR (400 MHz) of 1,4-diphenylpiperazine (**7**) in $CDCl_3$.

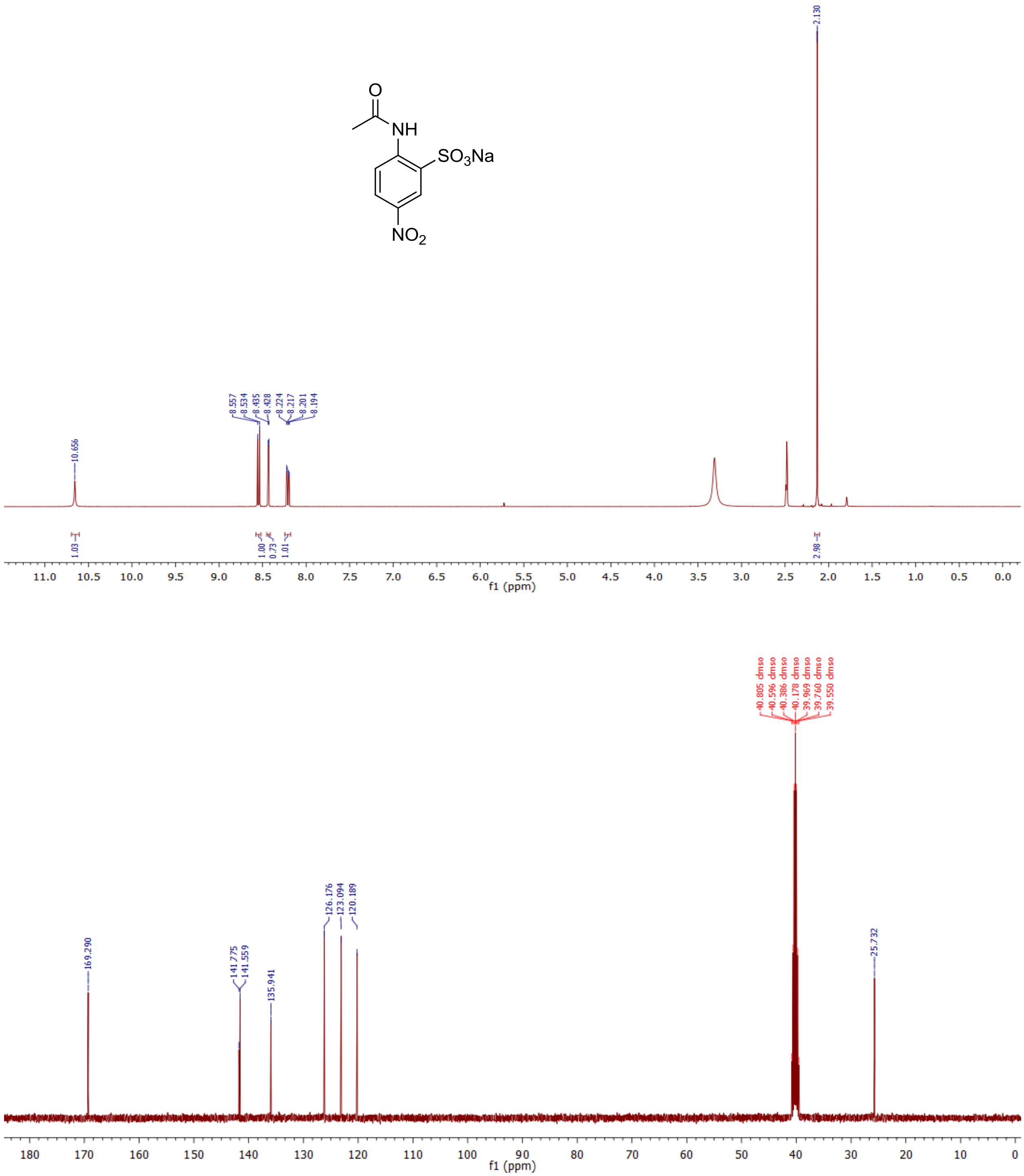

Figure S2: ^{1}H NMR (400 MHz) and ^{13}C NMR (100 MHz) spectra of sodium 2-acetamido-5-nitrobenzenesulfonate (**5**) in DMSO-d_6.

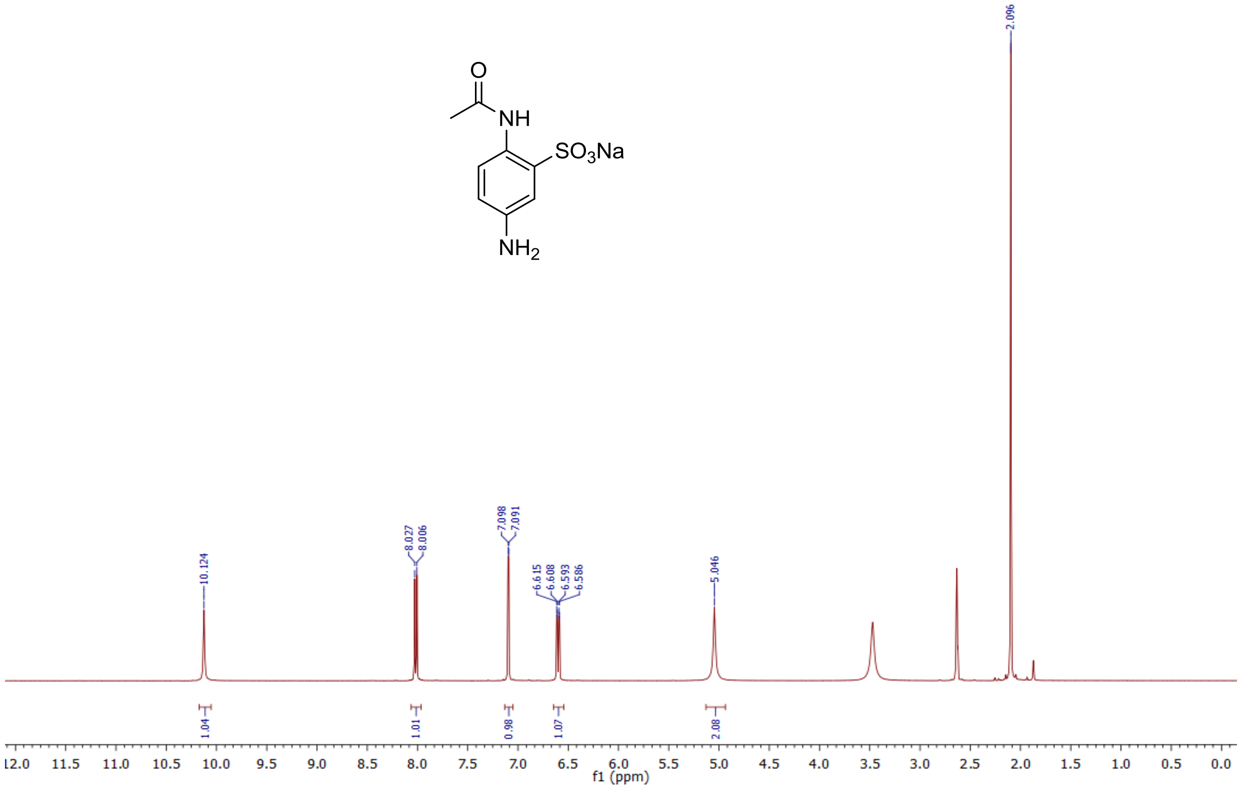

Figure S3: ^{1}H NMR (400 MHz) of sodium 2-acetamido-5-aminobenzenesulfonate (**4**) in DMSO-d_6.

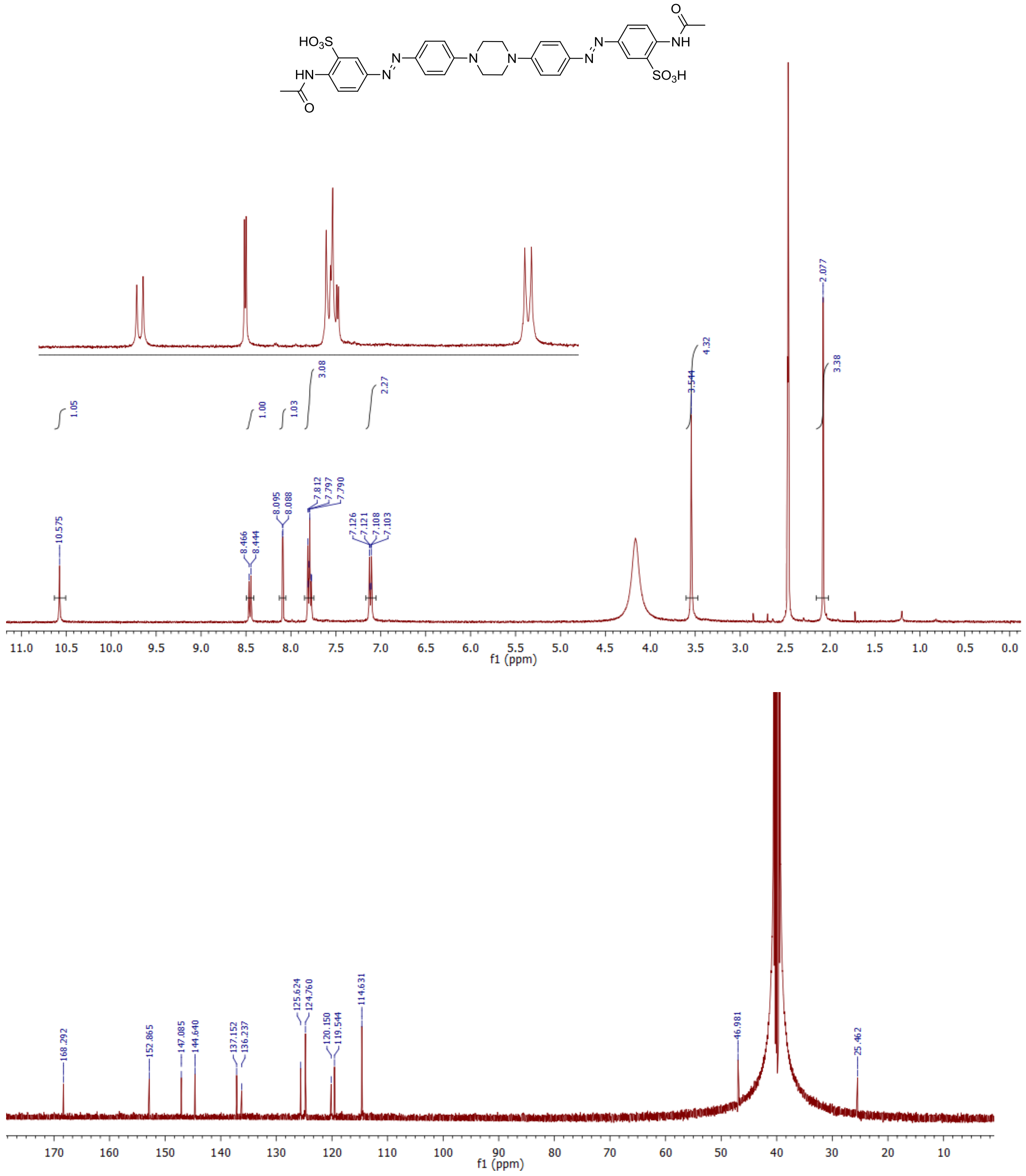

Figure S4: ^{1}H NMR (400 MHz) and ^{13}C NMR (125 MHz) spectra of 5,5'-(piperazine-1,4-diylbis(4,1-phenylene))bis(diazene-2,1-diyl)bis(2-acetamidobenzenesulfonic acid) (**2**) in DMSO-d_6.
